# Atrial Fibrillation Ablation After Three Decades: Mechanistic Insight or Just a Technological Race?

**DOI:** 10.3390/jcm14186601

**Published:** 2025-09-19

**Authors:** Giulia Spiriti, Antonio Scarà, Alessio Borrelli, Federico Zanin, Leonardo Pignalosa, Lorenzo Buzzelli, Zefferino Palamà, Antonio Gianluca Robles, Martina Nesti, Luigi Sciarra

**Affiliations:** 1 Unit of Cardiac Electrophysiology, San Carlo di Nancy Hospital, 00165 Rome, Italy; dott.giuliaspiriti@gmail.com (G.S.); alessioborrelli@libero.it (A.B.); federicozanin@msn.com (F.Z.); leonardopignalosa@hotmail.com (L.P.); 2 Department of Life, Health and Environmental Sciences, University of L’Aquila, 67100 L’Aquila, Italy; lorenzobuzzelli@hotmail.it; 3 Unit of Cardiology, Casa di Cura Villa Verde, 74121 Taranto, Italy; zefferino.palama@icloud.com; 4 Department of Cardiology, Ospedale “L. Bonomo”, 70031 Andria, Italy; gianlucarobles24@gmail.com; 5 Unit of Cardiology, CNR Fondazione Toscana “Gabriele Monasterio”, 56124 Pisa, Italy; martina.nesti@tiscali.it

**Keywords:** atrial fibrillation, energy sources, tailored approach

## Abstract

Atrial fibrillation (AF) is the most common sustained supraventricular arrhythmia, affecting 2–3% of the adult population and contributing significantly to morbidity, mortality, and healthcare burden. Catheter ablation has become a cornerstone in the treatment of symptomatic, drug-refractory AF, with pulmonary vein isolation (PVI) established as the standard approach, especially in paroxysmal AF. Over the past three decades, ablation technologies have evolved considerably—from radiofrequency and cryoballoon to the recent advent of pulsed field ablation—enhancing procedural safety, efficiency, and lesion durability. Despite these technological advancements, long-term outcomes have plateaued, suggesting that success may depend not just solely on the energy source used, but also on a more individualized, mechanism-based approach. The classification of AF based on duration alone fails to capture the complexity of its underlying pathophysiology. Tailored strategies that consider arrhythmic mechanisms, electrophysiological triggers, and patient-specific substrates—especially in persistent AF—are increasingly recognized as essential for durable results. Tools such as high-density mapping, autonomic modulation, and substrate-targeted ablation are expanding therapeutic horizons. Moreover, special populations, such as athletes, present unique arrhythmic profiles influenced by structural and autonomic remodeling, requiring nuanced management. The integration of lifestyle interventions, neuromodulation techniques, and emerging genetic and pharmacological insights further supports a comprehensive, personalized approach. In this paper, we explore whether future success in AF ablation lies more in refining technology or in advancing our understanding of arrhythmic mechanisms to guide patient-specific therapy.

## 1. Introduction

Atrial fibrillation (AF) is the most common sustained supraventricular arrhythmia, characterized by disorganized atrial electrical activity and ineffective contraction. It affects 2–3% of the adult population, with prevalence expected to double by 2060 due to demographic aging and improved diagnostics [1]. Clinically, AF is associated with symptoms such as palpitations, fatigue, and dyspnoea, and can lead to serious complications including stroke, heart failure, cognitive decline, and increased mortality. These outcomes contribute to a growing healthcare burden, both clinically and economically [2].

Catheter ablation has become an established therapy for symptomatic, drug-refractory AF, with effectiveness varying depending on the AF subtype. AF is currently classified based on duration—paroxysmal, persistent, and permanent—which informs treatment strategies. While ablation is rarely performed in permanent AF, pulmonary vein isolation (PVI) is most effective in paroxysmal cases.

The seminal work by Haïssaguerre et al. in 1999 identified pulmonary vein ectopy as a key AF trigger, establishing PVI as the cornerstone of modern ablation [3]. Since that initial discovery, a wide range of ablation tools and techniques have been developed to perform PVI, improving procedural safety, lesion durability, and long-term outcomes.

Currently, PVI can be achieved using three FDA- and CE-approved technologies: radiofrequency ablation (RFA), cryoballoon ablation (CBA), and pulsed field ablation (PFA). Each modality presents specific advantages and limitations in terms of procedural efficiency, safety, and durability.

Despite these technological advancements, mid- and long-term success rates appear to have plateaued. This raises the still-unresolved question of whether long-term outcomes are primarily determined by the ablation technology itself or by the strategic approach guiding its use [4].

In this paper, we aim to explore whether the cornerstone of effective AF ablation lies in the choice of technology per se, or a tailored approach based on each patient’s electrophysiological profile, arrhythmic triggers, and structural substrate. In other words, optimal outcomes may depend on selecting the right intervention for the right patient, shifting the focus from tools to a targeted, mechanism-driven therapy.

This perspective represents a synthesis of current expert understanding and interpretation. The recommendations presented should be viewed in light of the available evidence at the time of writing. In areas where data remain limited or are still evolving, these suggestions are offered as tentative considerations, open to revision as further evidence emerges.

## 2. Atrial Fibrillation Ablation

Notably, catheter ablation has become a cornerstone of AF therapy, particularly in symptomatic patients, with contemporary guidelines endorsing its use as a first-line treatment for paroxysmal AF (Class I, Level B) and as a second-line approach for persistent AF (Class IIb, Level C) [1]. Recent trials, such as CABANA and EAST-AFNET4, have demonstrated that early rhythm control, particularly via ablation, may improve outcomes and potentially prevent atrial remodeling [5,6].

Here is a brief analysis of the various technologies and energy sources now available for the ablation of AF.

### 2.1. Radiofrequency Ablation

Radiofrequency ablation (RFA) was the first energy source introduced into clinical practice for atrial fibrillation (AF) treatment and remains the most widely used modality today. Its mechanism of action is based on thermal injury leading to coagulative necrosis of the targeted myocardial tissue [7]. The historical foundation of RFA lies in the seminal work of Haïssaguerre et al., who identified pulmonary veins (PVs) as primary triggers of AF and demonstrated the efficacy of fluoroscopy-guided segmental ablation at the PV ostia [3]. This laid the groundwork for subsequent advances such as the integration of three-dimensional electroanatomic mapping systems. Pappone et al. pioneered the use of magnetically guided RF catheters to perform circumferential PV isolation (PVI), allowing for more complete and anatomically tailored lesion sets with reduced fluoroscopy exposure [8,9].

Technological advancements in RFA have significantly improved procedural safety and efficacy. Catheters have evolved from non-irrigated, temperature-controlled models to irrigated, power-controlled systems (20–40 Watts), effectively reducing thromboembolic risks [10]. The introduction of contact-force sensing catheters enabled the development of lesion quality indices—such as Ablation Index (AI, Biosense Webster) and Lesion Size Index (LSI, Abbott)—to standardize lesion creation and ensure tissue safety [11]. Remote catheter navigation systems, including robotic and magnetic technologies, have also been explored to enhance catheter stability, though outcomes have been variable [12].

The use of 3D-mapping systems with auto-tagging capabilities has led to standardized workflows such as the CLOSE protocol, designed to create continuous, optimized lesions and reduce PV reconnection rates [13]. With these developments, modern point-by-point RFA has become a safe and efficient strategy, often completed within an hour, achieving up to 98% first-pass isolation and 90% durability at one year.

The clinical efficacy of RFA has been validated in several randomized controlled trials (RCTs) comparing it to antiarrhythmic drug therapy (ADT), particularly in patients with paroxysmal AF. RFA consistently demonstrated superior outcomes in terms of arrhythmia recurrence and overall AF burden [14].

Despite its proven efficacy, RFA is associated with a spectrum of procedural risks. The majority of complications are acute and primarily involve vascular access issues, such as femoral pseudoaneurysms or bleeding, which are usually self-limited. More serious, though less frequent, complications include pericardial effusion, cardiac tamponade, and PV stenosis. One of the most feared, albeit rare, complications is atrioesophageal fistula, which carries a mortality rate of 70–80% if not promptly recognized and treated [15].

In conclusion, RF ablation remains a cornerstone of AF management due to its versatility, long-term efficacy, and continuous technological refinement. It holds a particularly advantageous position in substrate modification and tailored ablation strategies, especially in persistent AF. As innovations continue to enhance safety and efficiency, RFA is poised to maintain its central role within the electrophysiological armamentarium.

### 2.2. Cryoballon Ablation (CBA)

Cryoballoon ablation (CBA) represents the second most widely adopted energy source for atrial fibrillation (AF) ablation, following radiofrequency. This technique employs the rapid expansion of nitrous oxide gas within a balloon catheter to reduce tissue temperature to approximately –40 to –50 °C, inducing cellular necrosis via cryothermal injury. Since its initial introduction in 2003, CBA has undergone significant technological refinement, culminating in the widespread adoption of the second-generation cryoballoon system around 2012 [16].

Cryoablation has gained traction among electrophysiologists due to its simplified, single-shot approach and favorable operator learning curve [17]. Clinical trials have consistently demonstrated the non-inferiority of CBA compared to radiofrequency ablation (RFA), prompting its inclusion in international guidelines as a valid first-line option for PVI in patients with paroxysmal AF [18]. A landmark randomized controlled trial (RCT) established CBA as a safe and effective alternative to antiarrhythmic drug therapy (ADT) in symptomatic patients with paroxysmal or persistent AF who had failed at least one antiarrhythmic agent [19]. Subsequent trials, such as STOP-AF First and EARLY-AF, extended these findings by supporting CBA as an initial therapy, demonstrating superior arrhythmia-free survival and reduced AF burden through continuous rhythm monitoring [20,21]. Moreover, long-term data over a 3-year follow-up suggested that early ablation with cryoballoon may delay disease progression by reducing the transition to persistent AF or recurrent atrial tachyarrhythmias [22].

From a safety standpoint, CBA shows a favorable risk profile. Rates of major complications are comparable to those observed with ADT, with the EARLY-AF trial even reporting a slightly lower incidence of serious adverse events in the ablation arm (3.2% vs. 4%) [20]. Unlike RFA, cryoablation is associated with a negligible risk of atrioesophageal fistula and a reduced incidence of pericardial complications [15]. However, transient phrenic nerve palsy remains the most frequent procedural adverse event, though it typically resolves without long-term sequelae.

Currently, two main cryoballoon platforms are commercially available. The Arctic Front Advance Pro (Medtronic, Dublin, Ireland), now in its fourth generation, comes in 23 mm and 28 mm sizes and is characterized by improved cooling profiles and catheter stability. A newer entrant, the POLARx FIT (Boston Scientific, Marlborough, MA, USA), features an adjustable balloon diameter (28–31 mm) and an enhanced sheath deflection angle (155° vs. 135° of the Arctic Front), offering greater adaptability during challenging anatomies [23]. Additionally, innovations such as ultra-low temperature cryoablation (ULTC, Adagio Medical, Laguna Hills, CA, USA) are being explored. These linear catheters, equipped with shape-modifiable stylets, are capable of creating deeper lesions, but may be associated with increased risk of off-target damage.

### 2.3. Pulse Field Ablation (PFA)

In recent years, pulsed field ablation (PFA) has emerged as a major innovation in the treatment of atrial fibrillation (AF), following its clinical introduction in 2018. Unlike conventional thermal ablation techniques, such as radiofrequency ablation (RFA) and cryoballoon ablation (CBA), PFA is a nonthermal modality that employs short-duration, high-voltage electrical pulses to induce irreversible electroporation, resulting in targeted myocardial cell death without heat generation [24].

This unique mechanism allows for the selective ablation of atrial myocardium while minimizing injury to adjacent structures, particularly the esophagus, phrenic nerve, and pericardium. Indeed, preclinical and early clinical data consistently demonstrated a favorable safety profile, including the absence of esophageal injury on cardiac magnetic resonance imaging following PFA [25]. Nevertheless, recent reports have identified potential risks such as coronary vasospasm and hemolysis-related acute kidney injury, particularly during high-dose energy delivery [26,27,28]. The underlying mechanisms are thought to involve transient vascular smooth muscle contraction for vasospasm and red blood cell membrane disruption for hemolysis. Acute kidney injury is typically secondary to hemolysis-induced pigment nephropathy, where the release of free hemoglobin and heme leads to tubular injury and oxidative stress. Hemolysis is nearly universal following PFA but is usually subclinical, whereas clinically significant AKI occurs in approximately 0.05% of cases, and coronary vasospasm has been reported in about 0.14% of patients [28,29,30]. Despite these rare complications, the overall incidence remains low, as demonstrated in the aforementioned studies, and PFA continues to be a relatively new technology. Consequently, in the authors’ opinion, there is currently insufficient data to reliably guide patient selection based on the risk of these rare complications. Continued accumulation of safety and outcome data will be essential to refine selection criteria in the future.

Device-specific factors, including catheter design (single-shot vs. point-by-point) and pulse delivery protocols, may influence efficacy and complication patterns. Moreover, early clinical experience indicates that operators face a distinct learning curve with PFA, requiring specific training to optimize procedural efficiency and minimize complications compared with conventional thermal ablation techniques [31].

Furthermore, real-world experience has revealed platform-specific safety signals, such as transient pauses in device availability related to neurovascular events, underscoring the importance of vigilant surveillance and the distinct learning curve associated with PFA compared to thermal ablation systems [32]. One of the theoretical advantages of PFA lies in its ability to generate deeper and more homogeneous transmural lesions, especially in areas of thickened or fibrotic myocardium, which are often difficult to treat effectively with thermal approaches [25].

Preliminary single-arm studies suggest acute success rates of approximately 85–90% in paroxysmal AF, accompanied by improvements in quality of life and reductions in antiarrhythmic drug use, cardioversions, and hospitalizations [33,34].

However, reliance on uncontrolled cohorts limits the generalizability of these findings. More recently, randomized head-to-head trials have strengthened the evidence base: the ADVENT trial demonstrated that PFA (Farapulse system) was noninferior to conventional RFA/CBA for freedom from treatment failure at 12 months, with comparable rates of serious adverse events [25]. Similarly, a trial with continuous rhythm monitoring reported PFA to be noninferior to cryoballoon ablation in paroxysmal AF [35].

In persistent AF, early prospective studies, such as PULSED AF, indicate feasibility and safety, but definitive comparative data remain limited, and long-term durability beyond one year is still under investigation [33].

Taken together, while PFA shows promise as a safe and effective alternative to thermal ablation—particularly in paroxysmal AF—its role in persistent AF and its long-term outcomes require further validation through ongoing randomized controlled trials.

## 3. Tailored Therapy

**Figure 1:**
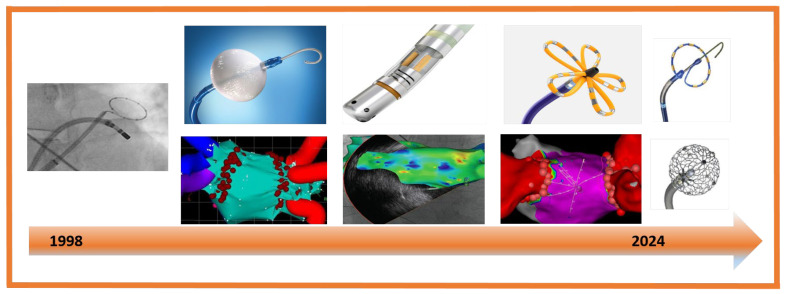
Contemporary techniques and energy sources for catheter ablation of atrial fibrillation.

**Scheme 1:**
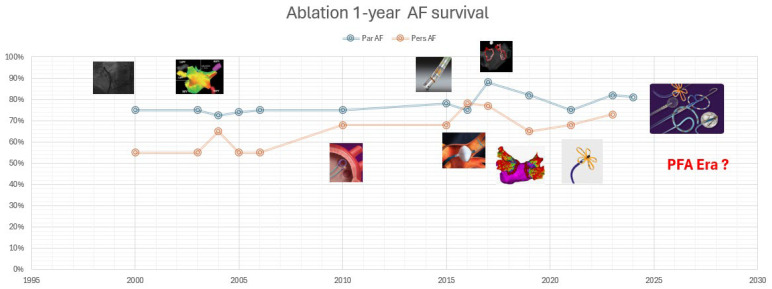
One-year freedom from atrial fibrillation recurrence following catheter ablation in paroxysmal and persistent AF across pivotal studies.

Despite substantial advancements in catheter ablation techniques, current outcomes in AF management appear to have plateaued (Figure 1, Table 1, and Scheme 1). The standardized approach commonly employed—where patients are referred directly for ablation without thorough pre-procedural assessment—limits the potential benefits of these advanced technologies. AF is multifactorial, with clinical manifestations varying greatly across individuals. Factors such as age, comorbidities (e.g., hypertension, diabetes, obesity), and autonomic tone influence the pathophysiology and progression of AF. Notably, the temporal classification of AF (paroxysmal versus persistent) does not always reflect the underlying mechanisms or predict its progression [14].

Admittedly, in carefully selected patients with paroxysmal AF and well-defined PV triggers, a standardized PVI-first strategy is both efficient and evidence-based. Randomized trials—including EARLY-AF (cryoballoon vs. antiarrhythmic drugs) [20], RFA-first trials such as MANTRA-PAF with favorable long-term outcomes [48], and RCTs like RAAFT-2 and meta-analyses showing superiority of RFA over drug therapy [49]—support this approach. However, outside this subgroup, a stepwise, individualized approach remains essential, encompassing detailed clinical evaluation and, when necessary, pre-ablation electrophysiological studies. These evaluations are critical for identifying arrhythmogenic mechanisms such as non-pulmonary vein triggers, thereby enabling a more personalized and effective treatment strategy.

**Figure 2:**
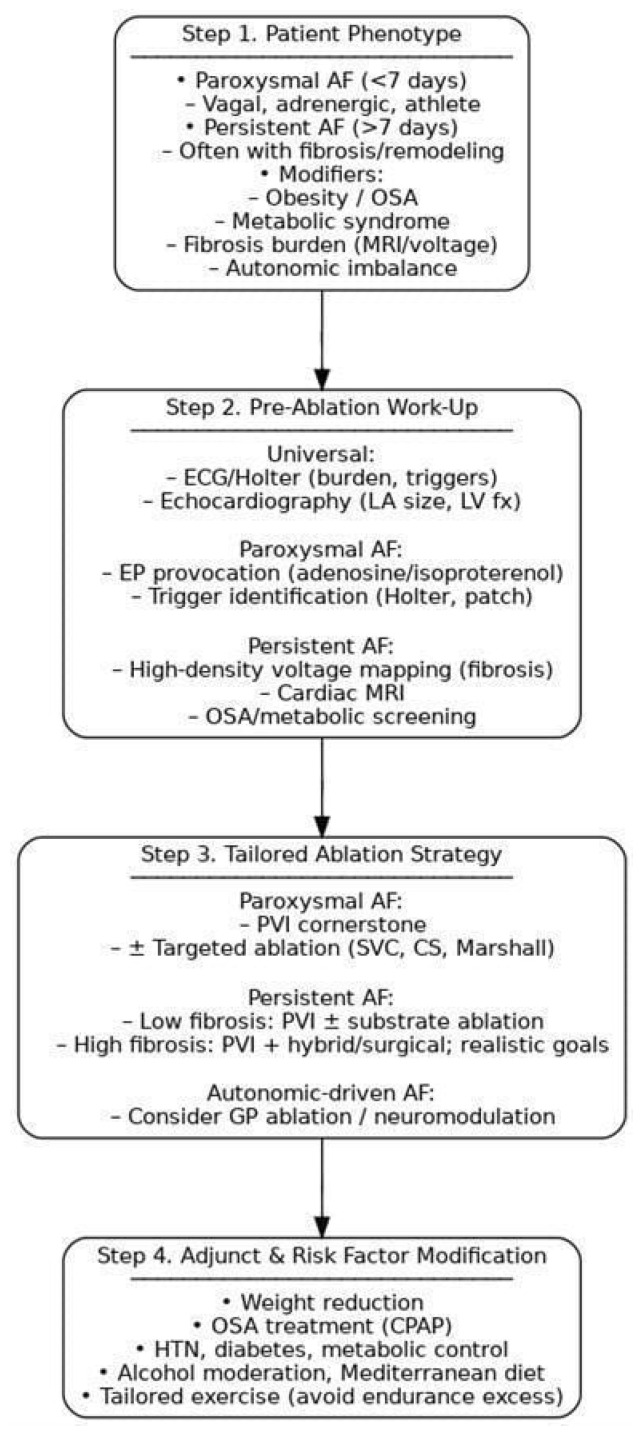
Stepwise approach to patient-tailored atrial fibrillation ablation, including phenotype assessment, pre-ablation work-up, procedural strategy, and risk factor modification.

As illustrated in the boxed algorithm (Figure 2), this approach emphasizes the importance of a careful preoperative assessment—including evaluation of patient phenotype, comorbidities, and atrial substrate—to guide individualized ablation strategies (Table 2) and adjunctive therapy choices.

### 3.1. Paroxysmal AF: Targeting Ectopic Triggers

Paroxysmal AF is characterized by episodes that last less than 7 days (usually 24–48 h) and typically resolve spontaneously. This self-limiting nature suggests that ectopic atrial triggers, often originating from the pulmonary veins (PVs), are the primary mechanism. Haissaguerre et al.’s seminal 1998 study, which identified the PVs as the predominant source of ectopic beats in paroxysmal AF, revolutionized ablation strategies with pulmonary vein isolation (PVI) [3]. The anatomical connection between the PVs and the left atrium, particularly at the venous-atrial junction, is arrhythmogenic due to electrical and histological discontinuities, which promote micro-reentry mechanisms [50]. Therefore, PVI remains the cornerstone of ablation for paroxysmal AF.

In addition to PVs, ectopic triggers may originate from other regions, including the right atrium, superior vena cava, coronary sinus, and the Marshall vein. Identifying these triggers through clinical evaluation and advanced diagnostic tools, such as continuous Holter monitoring or invasive electrophysiological studies, is crucial for a successful ablation [51,52].

Personalized strategies, such as identifying and targeting specific ectopic foci, have been shown to achieve success rates greater than 90% in highly selected paroxysmal AF patients with discrete PV triggers [2]. However, more recent randomized evidence, such as the ADVENT trial, reported 12-month arrhythmia-free survival rates of ~73% following pulsed field or thermal ablation, reflecting outcomes in broader real-world populations [25]. A tailored approach therefore requires careful pre-procedural analysis to avoid unnecessary and potentially harmful lesions, which underscores the importance of a thorough patient evaluation before PVI. Although time-consuming, this approach has demonstrated superior outcomes compared to empirical, non-targeted ablation [34].

### 3.2. Persistent AF: Substrate Modification

Persistent AF, on the other hand, involves a more complex pathophysiology and often requires a deeper understanding of the atrial substrate. Unlike paroxysmal AF, where triggers dominate, persistent AF is influenced by substrate changes that make the arrhythmia more refractory to conventional treatments [53]. Substrate modification techniques beyond PVI have been explored, but have shown variable success [54,55]. Studies have demonstrated that targeting complex fractionated atrial electrograms (CFAEs) or rotors and adding linear lesions may not provide additional benefits when compared to PVI alone. The progression of the disease, such as the development of atrial fibrosis or other anatomical changes, complicates the treatment of persistent AF, and the effectiveness of these strategies has yet to be conclusively proven [34].

Recent technological advancements, including high-density endocardial voltage mapping with multipolar catheters, allow for more precise identification of low-voltage areas in the left atrium, which are considered markers of atrial fibrosis [55]. These low-voltage zones are believed to harbor arrhythmogenic mechanisms in persistent AF and are targets for ablation. However, PVI remains the cornerstone of treatment, and substrate modification alone is insufficient in achieving long-term success [56]. Therefore, integrating both PVI and substrate-based approaches offers the potential for a more comprehensive and personalized treatment strategy.

In the setting of persistent atrial fibrillation, the concomitant presence of heart failure is not uncommon. In such scenarios, in addition to catheter ablation, lifestyle modification [57] may represent a key therapeutic component, and selected supportive pharmacological treatments may also provide clinical benefit. Recent meta-analyses, in fact, suggested that pharmacological interventions could potentially reduce the risk of new-onset atrial fibrillation (AF) in patients with heart failure (HF). Angiotensin-converting enzyme inhibitors and angiotensin receptor blockers (ACEIs/ARBs) have been shown to lower AF incidence, particularly in heart failure with reduced ejection fraction (HFrEF), with relative risk reductions (RR) approaching 0.57 in this subgroup [58]. Additionally, semaglutide, a GLP-1 receptor agonist, demonstrated a potential protective effect against AF onset (RR ≈ 0.60) [58]. Importantly, a more recent meta-analysis targeting sodium–glucose co transporter 2 inhibitors (SGLT2i) across various cardiovascular populations—including HF—reported a potential preventive effect against atrial fibrillation, further supporting the evolving role of HF-specific therapies in AF prevention [59].

### 3.3. Autonomic Nervous System and Atrial Fibrillation: Translational Therapeutic Perspectives

The role of the autonomic nervous system cannot be excluded from a comprehensive understanding of the mechanisms of atrial fibrillation. The interaction between sympathetic and parasympathetic inputs creates electrophysiological conditions that facilitate atrial ectopy and reentry mechanisms. Vagal stimulation, through muscarinic acetylcholine receptors, reduces action potential duration and atrial refractoriness, favoring reentrant circuits, while sympathetic activation increases intracellular calcium loading and promotes delayed afterdepolarizations, enhancing triggered activity. These phenomena are amplified by fluctuations in autonomic tone, which have been observed before AF onset in experimental and clinical studies [60,61].

Dynamic factors exert a profound influence on autonomic balance and AF burden. Conditions such as obesity, obstructive sleep apnea (OSA), alcohol intake, and gastroesophageal reflux contribute to atrial substrate remodeling through metabolic and inflammatory pathways, alongside modulation of autonomic inputs [62]. Epicardial fat, which hosts ganglionated plexi (GP), represents an important anatomical and functional link between adipose tissue and neural regulation [63]. Lifestyle-related factors, including diet composition and endurance exercise, further modulate ANS activity, increasing susceptibility to AF in predisposed individuals.

Therapeutic strategies have been developed to target ANS dysfunction either directly or indirectly. GP ablation, performed through selective high-frequency stimulation guidance or anatomical approaches, has shown variable success in reducing AF recurrence [64]. Right atrial ablation targeting specific GP clusters offers a potentially safer approach in selected cases. The combination of pulmonary vein isolation with GP ablation appears to enhance procedural efficacy, although concerns regarding reinnervation persist [64]. Minimally invasive surgical techniques incorporating GP ablation have not demonstrated significant additional benefit in advanced AF, as reported in the AFACT trial [65]. Recently, cardioneuroablation guided by spectral mapping has emerged as a promising approach to selectively target parasympathetic neural elements while minimizing collateral damage.

Non-direct neuromodulation techniques, including renal sympathetic denervation, stellate ganglion ablation, and low-level vagal nerve stimulation, have produced encouraging experimental and early clinical results, although long-term efficacy and safety remain to be established [66]. Concurrently, the correction of dynamic factors through weight reduction, OSA treatment with continuous positive airway pressure, and adherence to cardioprotective dietary patterns such as the Mediterranean diet is essential to achieve lasting control of autonomic imbalance and reduce AF recurrence [67,68].

Future perspectives point toward the integration of pharmacological, genetic, and interventional strategies. Novel antiarrhythmic agents targeting IK, ACh, and SK channels, along with modulation of calcium-handling proteins, offer the potential to influence autonomic-driven arrhythmogenesis. Advances in gene therapy and the concept of “ablatogenomics” may enable individualized ablation strategies based on genetic predisposition, with polymorphisms, such as those on chromosome 4q25, already implicated in ablation outcomes [69].

### 3.4. Atrial Fibrillation in a Specific Population: Athletes

When assessing patient characteristics, physical activity level is a critical factor. While moderate exercise reduces cardiovascular risk, prolonged high-intensity training increases AF incidence, particularly in middle-aged men—a phenomenon termed the “athlete’s paradox,” reflecting cardiovascular benefits alongside elevated arrhythmic risk [70,71].

AF in athletes arises from structural, electrical, and autonomic adaptations of the “athlete’s heart.” Chronic endurance exercise induces biatrial enlargement, atrial wall stretch, and occasionally myocardial fibrosis. Although dilation typically preserves compliance, sustained hemodynamic stress promotes an arrhythmogenic substrate [72].

Autonomic remodeling is central: endurance training enhances parasympathetic tone and attenuates sympathetic drive at rest, producing sinus bradycardia and increased heart rate variability. Vagal predominance shortens atrial refractoriness, increases repolarization heterogeneity, and favors reentry [73]. AF episodes commonly occur during recovery or sleep, while adrenergic AF during exertion is rare.

Triggers frequently originate from pulmonary veins, as in the general population, although athletes may exhibit greater atrial ectopy. Experimental models confirm that exercise-induced remodeling heightens AF inducibility [74].

Clinically, AF in athletes is usually paroxysmal, manifesting as palpitations, reduced performance, or exertional fatigue. Despite lower overall mortality, AF impairs eligibility for competitive sports. Pharmacological rhythm control is limited by bradycardia and anti-doping concerns, making catheter ablation the preferred strategy in symptomatic cases [75].

Sex-related differences in atrial fibrillation (AF) incidence among athletes are complex and are not fully elucidated. While evidence suggests a generally lower risk in women, AF risk in female endurance athletes remains poorly quantified and may vary according to sport type, training load, and hormonal influences [52,76]. AF susceptibility in athletes reflects a multifactorial interplay of sex, training intensity, and genetic predisposition, underscoring the need for individualized assessment and management [76,77].

Following catheter ablation, return-to-play decisions should be guided by structured monitoring and personalized evaluation, [70,77]. The 2024 HRS Expert Consensus Statement on Arrhythmias in the Athlete recommends a structured approach to return-to-play decisions, including a thorough risk assessment and shared decision-making between the athlete and healthcare providers. This ensures that the athlete’s health and safety are prioritized while considering their desire to resume athletic activities [78].

## 4. Conclusions

In conclusion, the management of AF, particularly paroxysmal and persistent forms, requires a personalized and tailored approach. The role of catheter ablation, whether via radiofrequency, cryoballoon, or pulsed field ablation, should be carefully considered based on the patient’s specific arrhythmic mechanisms, comorbidities, and procedural goals. Although PVI remains central to treatment, emerging technologies such as high-density mapping, rotor and focal activity ablation, and hybrid approaches are offering new avenues for addressing persistent AF. Furthermore, our evolving understanding of AF substrate modification and the integration of advanced mapping technologies enable more precise and individualized treatments, improving outcomes and reducing the risk of complications. The future of AF management will likely revolve around a combination of advanced electrophysiological techniques, careful pre-procedural assessment, and a multidisciplinary approach to ensure optimal, patient-centered care.

## Figures and Tables

**Table 1:** Outcomes of catheter ablation in atrial fibrillation: freedom from recurrence in paroxysmal and persistent AF across major studies.

Year	Par AF	Pers AF	Study
2000	75%	n/a	Haïssaguerre et al. (Circulation) [36]
2003	75%	55%	Oral et al. (Circulation) [37]
2004	73%	55%	Jais et al. (Circulation) [38]
2005	74%	65%	Lang et al. (JACC) [39]
2006	75%	55%	Chen et al. (JACC) [40]
2010	75%	55%	Verma et al. [41]
2015	78%	68%	Reddy et al. (JACC: Clinical EP) [42]
2016	75%	68%	Fire and ICE [43]
2018	88%	78%	Close [44]
2019	82%	77%	Reddy et al. (JACC) [45]
2023	75%	65%	ADVENT [25]
2023	82%	68%	Musikantow et al. (JACC EP) [46]
2024	81%	73%	QDOT MICRO REGISTRY [47]

**Table 2:** Clinical scenarios in atrial fibrillation: dominant mechanisms, ablation strategies, and energy source considerations.

Clinical Scenario	Dominant Mechanism	Preferred Lesion Strategy	Energy Considerations
Symptomatic paroxysmal AF, structurally normal atria	PV triggers	Wide antral PVI	CBA or PFA for efficiency; RFA for anatomy variants
Paroxysmal AF with suspected non-PV triggers	Focal ectopy	Map + focal ablation ± PVI	RFA (versatile); focal PFA emerging
Early persistent AF, mild substrate	PV + limited substrate	PVI ± targeted LVA/driver ablation	RFA (tailored); investigational PFA
Advanced persistent AF, atrial dilation/fibrosis	Substrate-dominant	PVI + voltage-guided homogenization ± lines ± hybrid posterior wall	RFA backbone; add surgical/hybrid; PFA future
Vagal AF phenotype	Autonomic modulation	PVI ± GP ablation; lifestyle	RFA platform for GP; investigational neuromodulation
Endurance athlete	PV triggers + vagal modulation	Efficient PVI; selective adjuncts; return-to-play focus	CBA for speed; RFA for adjunct; PFA under study

## Data Availability

No new data were created or analyzed in this study.
